# Influence of outflow cannula geometry on hemodynamics in patients with partial circulatory support : A computational fluid dynamics (CFD)-study

**DOI:** 10.1186/1749-8090-10-S1-A95

**Published:** 2015-12-16

**Authors:** Jennifer Engelke, Aron-Frederik Popov, Sasan Partovi, Matthias Karck, Christof Karmonik, André R Simon, Fabian Rengier, Arjang Ruhparwar

**Affiliations:** 1 Department of Cardiac Surgery, University Hospital Heidelberg, Heidelberg, 69120, Germany; 2 Consultant Cardiac Surgeon, Royal Brompton & Harefield NHS Foundation Trust, Harefield, Middlesex, UB9 6JH, UK; 3 Department of Radiology, University Hospitals Case Medical Center, Case Western Reserve University, Cleveland, OH 44106, USA; 4 Translational Imaging MRI Core, Houston methodist Research Institute, Houston, TX 77030, USA; 5 Department of Cardiothoracic Transplantation and Mechanical Circulatory Support, Harefield Hospital, Royal Brompton and Harefield NHS Foundation Trust, London, UK; 6 Department of Radiology, University Hospital Heidelberg, Heidelberg, 69120, Germany

## Background/Introduction

Partial circulatory support is a new option of treatment for heart-failure patients. The Circulite Synergy Micro-Pump is a partial support device where the inflow cannula is connected to the left atrium and the outflow cannula to the right subclavian artery (RSA). Computational fluid dynamics (CFD) simulations can be used to demonstrate changes in hemodynamics post treatment.

## Aims/Objectives

To investigate the influence of the outflow cannula geometry on hemodynamics in the aorta and supra-aortic vessels in patients with a Circulite Synergy Micro-Pump.

## Method

Lumina of the aorta and supra-aortic vessels of nine patients were segmented from computed tomography angiographic (CTA) image data. Computational polyhedral meshes were created to conduct CFD-simulations (STAR CCM+) for diastolic flow conditions (turbulent flow model, diastolic heart flow velocity 0.25 m/s). To quantify effect of partial circulatory support, two simulations per case were carried out (with only native cardiac output and with the Circulite support device added, flow velocity 1 m/s).

Velocity magnitudes, velocities in inferior-superior direction (vz) and wall shear stresses were averaged in the innominate artery and descending aorta.

Cases were divided into two groups: Outflow cannula placed orthogonal to the RSA (n = 5, group 1) angle between the cannula and the RSA distal to the anastomosis site smaller than 90° (n = 4, group 2).

## Results

Geometry of the outflow cannula affected vz of the RSA and the innominate artery significantly (p < 0.05), but not in the descending aorta.

Increase of velocity in the descending aorta was similar in both groups (group1: 37.27%; group 2: 41.01%).

Wall shear stress in the innominate artery did not change in group 1(-6.06%), but increased in group 2(+334.12%). The difference was not significant.

## Discussion/Conclusion

Angle between SA and outflow cannula in patients with the Circulite Synergy Micro-Pump causes significant alterations in the subclavian and innominate arteries at diastole, but not in the descending aorta.

